# Associations between physical activity, screen time, sleep time and selected academic skills in 8/9-year-old children

**DOI:** 10.1186/s12889-023-16230-5

**Published:** 2023-07-12

**Authors:** Agata Korcz, Jana Krzysztoszek, Michał Bronikowski, Marlena Łopatka, Łukasz Bojkowski

**Affiliations:** 1 Department of Didactics of Physical Activity, Poznan University of Physical Education, Krolowej Jadwigi 27/39, Poznan, 61-871 Poland; 2 Department of Psychology, Poznan University of Physical Education, Krolowej Jadwigi 27/39, Poznan, 61-871 Poland

**Keywords:** Physical activity, Screen time, Sleep time, Academic skills, Primary school, Children

## Abstract

**Background:**

High levels of physical activity (PA), low levels of screen time, combined with sufficient sleep time, provide better health benefits. However, few studies have examined the association of these behaviours with academic skills. Therefore, this study aims to determine how PA, screen time, and sleep time are related to selected academic skills of 8/9-year-old children while examining compliance with the guidelines on PA, sedentary behaviour, and sleep among this population group.

**Methods:**

This cross-sectional study included 114 primary school children (50% girls) aged 8–9years old from 2nd grade. The levels of PA, screen time, and sleep were assessed using self-reported questionnaires. The selected academic skills (based on reading and writing) were assessed by a battery of methods designed to diagnose the causes of school failure in students aged 7–9. Non-linear regression was applied to build multivariate models aimed at finding the most significant predictors for the selected academic skills separately.

**Results:**

Sixty-seven percent of children met the sleep guidelines, 22% met the screen time guidelines, and only 8% met PA guidelines. In terms of screen time, boys spent more time playing games than girls (*p* = .008). Moderate to vigorous intensity physical activity (MVPA) was associated with higher/better scores of the visual-auditory integration (B = -0.07, *p* = .040). Not meeting the sleep guidelines was associated with lower scores in visual-auditory integration among children (B = 0.12, *p* = .042). Children who did not meet the screen time guidelines had lower scores in perceptual-motor integration (B = -0.09, *p* = .040).

**Conclusions:**

Participating in PA, limiting screen time and sufficient sleep time may benefit/support academic skills in children.

## Background

The sound development of a child is a fundamental objective in all education systems. This encompasses the stimulation of different aspects, including cognitive development for acquiring knowledge and intellectual potential, social development for acquiring skills needed to thrive in society, and motor development for enhancing fitness and PA skills needed for maintaining good health throughout life. However, the latest report on the state of children health published by World Health Organization (WHO) [1] identifies obesity and non-communicable diseases (side effects of reduced amount of PA) as significant health threats to children. These threats underline the need to address and prioritise the promotion of PA. Despite evidence highlighting the importance of daily PA for mental, social, and physical health [2–4], and the well-known benefits it offers, such as reducing the risk of cardiovascular diseases, mental health problems, and metabolic factors like obesity, hypertension, and diabetes [5–8], the prevalence of children achieving these guidelines remains alarmingly low [9].

In Poland, the results of the Report Cards for 2016 (Global Matrix, GM 2.0) and 2018 (GM 3.0) revealed a significant lack of PA among children and adolescents. According to the last assessment of PA (Report Cards for 2022, GM 4.0) of children and adolescents, three indicators slightly improved: Organised Sport and PA, Physical Fitness, and School. However, scores for Active Transport and Government were comparatively lower. No positive changes regarding Overall Physical Activity (OPA) or Sedentary Behaviours (SB) were observed. Moreover, in this edition, OPA was graded as incomplete due to methodological changes. However, if the previous WHO recommendations were used as a benchmark in GM 3.0, the score for the OPA indicator would decrease [10]. In the studies used a lower percentage of children and adolescents met the former WHO recommendations than in GM 3.0 [11].

The WHO recommends that children and adolescents aged 5–17years should accumulate at least 60min MVPA per day, including vigorous aerobic activities and muscle-and-bone-strengthening activities at least 3days per week [12]. Additionally, guidelines recommend limiting recreational screen time to no more than 2h per day and ensuring sufficient sleep (9 to 11h of sleep for children aged 5–13years) [13]. While the importance of the relationship of the abovementioned mediators of children’s overall health status has been already incorporated in research [14], their integration into educational and family settings for child development is still lacking. Thus, findings from innovative studies in this context are vital to provide effective solutions and a broader understanding of the topic.

However, thus far, schools, which should ideally be the most child-friendly and educative environment for such actions and where children spend most of their time, have been found to have limited effectiveness in this regard [15, 16].

This issue is compounded by the increasing impact of modern technology on the quality of health-related behaviours in children’s upbringing. The amount of time playing (not learning or working) on electronic devices, such as tablets, computers, and smartphones, has escalated beyond unreasonable levels [17], necessitating and calling for clear policies at national or even global levels to prevent further development. The longer the screen time, the more sedentary behaviour arises, which has long been recognised as a potential health hazard [18]. In fact, North American children and youth are spending 40–60% of their waking hours engaged in sedentary activities [19]. The problem got even worse with the outburst of the COVID-19 pandemic. Reports on health behaviours of children and youth indicate an alarming increase of screen time in this period [20]. This, in turn, has a huge impact on both the quality and duration of sleep among children and youth. A study by Dewald et al. [21] found strong associations between three sleep variables [sleep quality (*r* = 0.096), duration (*r* = 0.069), and sleepiness (*r* = -0.133)] and school performance, especially in boys. Yet, Brown et al. [22], analysing risky behaviours in a sample of 13,677 American adolescents in grades 9 through 12, discovered significant curvilinear relationships between PA, sleep, and academic achievement. Optimal grades were associated with 7–9h of sleep per night and 5–7h of PA per week. The study revealed that lower levels of PA were linked to poorer academic achievements. Interestingly, the interaction between the three key factors (sleep, PA, academic achievements) showed consistency across different levels of PA engagement, suggesting that PA alone does not independently impact academic achievements and that other movement behaviors may also play a mediating role. Similar interaction patterns have been observed between academic achievements and levels of fitness in previous studies [23]. Helping children move from the lower fitness levels to a higher percentile does not necessarily bring equally positive linear effects on academic achievements, but the authors [23] point that this could be contributed to the low robustness of PA measures (e.g., self-reported questionnaires).

Faught et al. [24] discovered a strong association between lifestyle behaviours (including PA, sleep, and screen time) and student academic performance. Their findings suggest that health promotion initiatives that target multiple lifestyle behaviours may have a greater impact on academic achievement compared to interventions focusing on a behaviour.

All the abovementioned factors are reflected in the child’s academic performance. Indeed, the relationship between PA, other daily time behaviours (e.g., screen time), cognitive functions, and academic learning has been receiving quite considerable amount of scientific interest [23, 25]. However, while some studies have found positive correlations between academic achievements and PA or fitness levels, these findings have primarily been obtained from cross-sectional and longitudinal studies. Whereas results from controlled experiments evaluating the specific benefits of PA on academic performance have been mixed and inconclusive [26]. Therefore, the association between PA and academic performance requires further investigation.

Thus, based on the abovementioned findings and evidence-based research, we have designed a study with the following objectives: 1) to determine how PA, screen time, and sleep time are related to selected academic skills of 8/9-year-old children, and 2) to examine the prevalence of adherence to the guidelines on PA, sedentary behaviour, and sleep for 8/9-year-old children.

## Methods

### Participants and study design

This cross-sectional study included 114 primary school children (57 girls and 57 boys) aged 8–9years old from 2^nd^ grade. All participants were a convenience sample of children from 4 primary schools and attended a standard primary school programme in the urban area of Poznan, Poland. This study was conducted in the winter of 2020 as part of a larger study/research activity “Active before the first bell” programme (research activity number 2019/03/X/NZ7/00313). A detailed information letter was given in paper form to parents or custodians for all participants. Approval of the child’s participation was assumed if the form was returned. Participation was voluntary, and the anonymity, as well as the confidentiality of the participants, were ensured. The study protocol was approved by the Local Bioethics Committee of The Karol Marcinkowski University of Medical Sciences in Poznan (decision number 729/19).

The assessments conducted in this study included body height and weight, self-reported PA, screen time, sleep time, and selected academic skills. Body mass and height data were collected by trained personnel with the use of an anthropological instrument (Wunder Sa. Bi. Srl., Italy). Body mass index (BMI) was calculated by dividing body weight with the squared height in meters (kg/m^2^). The participants were informed about the technique of the tests and each test item was accompanied by specific instructions presented to the participants. All measures and tests were performed in the schools (gym and classroom) and conducted in a friendly and comfortable atmosphere for the participant. All questionnaires were administered separately to children, where they were assisted on a “one-to-one” basis by a trained staff member and in comfortable setting for the child.

### Self-reported physical activity, screen time and sleep duration

PA, screen time, and sleep time were assessed using self-reported items that had been used previously in the Health Behaviour in School-aged Children (HBSC) study at national, regional, and international levels [27–29].

The level of MVPA was determined with a Physical Activity Screening Measure [30]. Participants were asked to answer two questions: 1) over the past 7days, on how many days were you physically active for a total of at least 60min per day? and 2) over a typical or usual week, on how many days are you physically active for a total of at least 60min per day? There were 8 response categories (0days, 1day up to 7days). The MVPA index was calculated based on the formula: MVPA = (Q1 + Q2)/2, where: MVPA represents the PA ratio, Q1 is the number of physically active days during the past 7days, and Q2 is the number of physically active days during typical week. According to Prochaska et al. [30], the measure is reliable (ICC = 0.77). Furthermore, this measure has also been used earlier in Polish studies as well [31–34]. Meeting the PA guideline was defined as engaging in at least 60min of MVPA every day. Therefore, in the present study, the time spent in MVPA was dichotomized as: (1) MVPA = 7days/week (recommended level), or (2) MVPA < 7days/week (below-recommended level).

Self-reported screen time was evaluated by three items: 1) “How many hours a day, in your free time, do you usually spend watching TV, videos (including YouTube or similar services), DVDs, and other entertainment on a screen?”; 2) “How many hours a day, in your free time, do you usually spend playing games on a computer, games console (like Playstation, Xbox, GameCube), tablet, smartphone or other electronic device (not including moving or fitness games)?” and 3) “How many hours a day, in your free time, do you usually spend using electronic devices such as computers, tablets or smart phones for other purposes, for example, homework, emailing, Facebook, Instagram, chatting, surfing the Internet?”. For each item, 9 response options were provided, ranging from ‘0’ to ‘around 7 or more than 7h’, separately for weekdays and weekends. The amount of screen time was reported by the respondent separately for school days (P1) and weekends (P2). A screen-time index was calculated based on the following formula: Screen time = (P1 × 5 + P2 × 2)/7, where Screen time = P1 = the number of hours per day on school days, P2 = the number of hours per day at the weekend. The screen time score is a weighted average of the entire week [33]. A cutoff at 2 h/day (≤ 2h per day and > 2h per day) was set to extract an additional dichotomised variable to reflect recommendations of the American Academy of Pediatrics [35], it was estimated only for watching TV and movies.

Sleep time during school days was determined by using two items: bedtime and wake-up time. Bedtime was measured with the item: “At what time do you usually go to bed when you are going to school the next morning?” and the response ranged in half-hour intervals from “No later than 21:00” to “2:00 or later”. Wake-up time was measured with the item: “At what time do you usually get up, when you are going to school?” Wake time responses ranged from “no earlier than 5:00” to “8:00 or later”. The American Academy of Sleep Medicine [36] recommends that 8–9-year-old children should sleep 9–12h regularly during a 24-h period for optimal health. Sleeping the recommended hours is associated with better health outcomes, while regularly sleeping fewer than the number of recommended hours is associated with numerous health and cognitive issues [36]. In this study, sleep durations less than 9h were coded as “insufficient”; sleep durations 9h and more as “sufficient”.

### A battery of methods for diagnosing the causes of school failure in students aged 7–9 (Battery-7/9)

Battery-7/9 [37] is intended for the psychological and pedagogical diagnosis of specific difficulties in reading and writing for children aged 7–9years. The tool covers the diagnosis of auditory and linguistic functions (awareness and phonological skills), visual-spatial functions (visual processing), perceptual-motor integration and the rate of naming visual stimuli. In terms of academic skills, it diagnoses the level of reading and writing [37]. The tasks that required time measurement were measured by stopwatches. Only selected tests were used from the Battery-7/9:

A) Figure Compare Test
  The purpose of the test is to assess pattern and spatial perception, the speed of visual perception and visual attention. The test evaluates seven patterns (geometric figures). The examined person is presented with a model figure, and below, six patterns, from which the participant has to indicate the same shape as the one presented above. The factors considered when assessing the results of the test are the correctness of responses to individual tasks and the total time of their completion. The minimum value of the index is 0 and the maximum value is 7. The value of the additional index depends on the participant's pace. The coefficient alpha for the Polish version is 0.41 [37].
B) Quick Naming Test
  The purpose of the test is to assess visual-auditory integration, long-term verbal memory, and rapid, automatic word recall. A set of three quick-naming tasks has been developed to evaluate the naming rate. The subject's task is to quickly name 50 colored stars (black, blue, green, red, yellow), 40 simple drawings (cheese, ice cream, sun, ship, clock, banana, etc.) and mixed pseudo-randomly 29 drawings and 19 letters. The indicator used in the task is the time taken to name all objects. The time is recorded for each task separately. Additionally, errors made by the participant are added to the time index (each error is an additional second). The coefficient alpha for the Polish version is 0.56 for colors, 0.74 for pictures and 0.75 for colors and pictures together [37].
C) Auditory-visual Integration Test
  The purpose of the test is to assess the ability to integrate multimodal and motor information and transfer information from one modality to another, as well as to assess memory. The examiner dictates successive rows of sounds, and the participant is required to write them down on a piece of paper. The analysis of psychometric properties includes the main indicator—the number of correct answers. The minimum value of the indicator is 0 and the maximum is 12. The coefficient alpha for the Polish version is 0.42 [37].
D) Selected Auditory-linguistic Test—Phonological Memory Test
  The purpose of the test is to assess the phonological aspect of the auditory-linguistic functions and the auditory memory of words. The moderator reads a series of words to the subject and asks the participant to recreate the series from their memory. The analysis of psychometric properties includes the main indicator, which is the number of correct answers. The minimum value of the indicator is 0 and the maximum is 18 [37]. The coefficient alpha was not calculated.

### Statistical analysis

The distribution of quantitative (continuous) variables, such as MVPA, screen time, sleep time and selected school skills, was verified using Shapiro–Wilk test. The quantitative (continuous) variables were presented as mean (M) and standard deviation (SD), while the qualitative (categorical) variables were presented as count (n) and percentage (%). Differences in baseline characteristics between girls and boys were assessed using the Mann–Whitney U test and/or T-test for continuous variables and the Chi-square test for categorical variables. For intergroup differences, the appropriate effect sizes were calculated depending on the test used [38]. Non-parametric tests were used in further analyses. The analyses were performed only for participants who had complete data for a given variable. The predictors were chosen based on the non-parametric tests Spearman correlation matrix, the Mann–Whitney U test, the Chi-square test, and literature analysis [39]. The selected predictors did not show significant correlation with each other, but they did correlate with the dependent variable. To determine the relationship between PA, screen time, sleep time, and academic skills, generalised non-linear models based on Wald statistics were performed. Raw models were made, followed by multi-factor models using the identity function. The following variables were included in the models: MVPA as a continuous variable, screen time in categories, sleep time in categories, gender. β coefficients with a "-" sign in relation to variables expressed in a time unit (sec) indicate a better result. The control group (reference points – ref.) consisted of girls and students who met the screen time and sleep time guidelines. The reference points were adopted as follows: MVPA (continuous variable), and sex (ref. female), screen time > 2h/day (ref. ≤ 2h/day) and sleep time < 9h/day (ref. ≥ 9h/day). All p-values *p* < 0.05 was considered statistically significant. Data were analysed using Statistica 13.1 (StatSoftPolska sp. z o.o., 2021).

## Results

A description of varying sample sizes as well as a description of behaviours (PA, screen and sleep time), can be seen in Table 1.

**Table 1:** Participants characteristics by sex mean and (SD) or n (%)

	Total n	Total	Girls	Boys	*p*	ES
Age (years)	114	8.6 (0.3)	8.63 (0.27)	8.62 (0.37)	.584^a^	0.31 ^D Cohen^
Hight (cm)	101	135.1 (6.1)	136.0 (6.2)	134.5.0 (6.1)	.320^a^	0.24 ^D Cohen^
Weight (kg)	101	32.1 ± 7.3	30.6 (5.7)	33.4 (8.3)	.065^b^	0.39 ^Glass^
BMI (kg/m^2^)	101	17.4 (2.9)	16.9 (2.4)	17.9 (3.3)	.058^b^	0.42 ^Glass^
MVPA (days/week)	101	3.3 (1.7)	3.5 (1.8)	3.2 (1.6)	.318^b^	0.17 ^Glass^
*MVPA categories*^*d*^	101					
MVPA = 7 days/week		8 (8%)	5 (10%)	3 (6%)	.444^c^	0.08 ^Phi^
MVPA < 7 days/week		93 (92%)	45 (90%)	48 (94%)		
Screen time (hrs/day)^e^	107	3.3 (1.6)	3.0 (1.2)	3.7 (1.9)	.193^b^	0.58 ^Glass^
Screen time (hrs/day)f	114	2.5 (1.7)	2.1 (1.4)	2.9 (1.8)	**.008**^**b**^	**0.57**^Gla**ss**^
Screen time (hrs/day)^g^	114	2.0 (1.3)	1.8 (1.0)	2.1 (1.6)	.400^b^	0.3 ^Glass^
*Screen time categories*^*h*^	107					
≤ 2 h/day		23 (22%)	12 (23%)	11 (20%)	.775^c^	0.28 ^Phi^
> 2 h/day		84 (78,5%)	41 (77%)	43 (80%)		
Sleep time (hrs/night)^i^	106	9.0 (0.9)	9.0 (0.9)	8.9 (1.0)	.972^c^	0.11 ^Glass^
*Sleep time categories*^*j*^						
< 9 h/day		39 (37%)	21 (40%)	18 (33%)	.452^c^	0.07 ^Phi^
≥ 9 h/day		67 (63%)	31 (60%)	36 (67%)		
*Selected academic skills*						
Visual-spatial functions (pts)	106	4.9 (1.5)	5.1 (1.4)	4.7 (1.4)	.137^b^	0.29 ^Glass^
Visual-spatial functions (sec)	106	82.2 (47.7)	90.2 (59.1)	74.6 (32.0)	.061^b^	0.29 ^Glass^
Visual-auditory integration – colors (sec)	106	52.8 (14.0)	55.4 (17.6)	50.4 (8.9)	.313^b^	0.28 ^Glass^
Visual-auditory integration –- drawings (sec)	106	46.1 (12.9)	46.8 (12.9)	45.3 (13.1)	.589^b^	0.12 ^Glass^
Visual-auditory integration – drawings/letters (sec)	106	55.9 (18.3)	58.7 (20.8)	53.2 (15.3)	.145^b^	0.26 ^Glass^
Auditory integration (sec)	111	147.8 (50.7)	152.2 (58.4)	143.6 (41.9)	.379^b^	0.15 ^Glass^
Perceptual-motor integration (pts)	99	6.3 (5.6)	7.4 (7.6)	5.3 (2.0)	**.013**^**b**^	**0.28**^Glass^
Auditory and linguistic functions (pts)	103	6.7 (2.5)	7.2 (2.5)	6.3 (2.3)	**.034**^**b**^	**0.33**^Glass^

^a^t-Student test

^b^Mann-Whitney U test

^c^Chi-squared test for sex differences, *ES* effect size, *D Cohen* Cohen’s d coefficient, Phi- Phi coefficient, Glass—Glass’s Delta coefficient. Bold indicates statistically significant results

^d^The MVPA level = 7 days means compliance with the WHO recommendations for the 5–17 age group

^e^Watching TV, videos (including YouTube or similar services), DVDs, and other entertainment on a screen

^f^Playing games on a computer, games console (like PlayStation, Xbox, GameCube), tablet, smartphone, or other electronic device (not including moving or fitness games)

^g^Using electronic devices such computers, tablets or smart phones for other purposes, for example, homework, emailing, Facebook, Instagram, chatting, surfing the Internet

^h^Screen time guidelines of less than 2 h of leisure screen activity

^i^Sleep time from reported usual bedtimes and wake times

^j^Sleep guidelines 9 to 12 h for children 5–13 years old

A total of 8% of children reported meeting PA guidelines of 60min on 7days per week. On average, children reported achieving at least 60min of PA on 3.3 ± 1.7days per week (see Table 1). When examined by sex, 10% of girls reported meeting PA guidelines, compared to 6% of boys (no significant differences were found).

Twenty-two percent of pupils met the screen time guidelines, and there were no statistical differences between girls and boys (23% vs 20% respectively). Boys reported watching TV, videos (including YouTube or similar services), DVDs, and other entertainment on a screen for an average of 3.7 ± 1.9h/day, while girls, 3.0 ± 1.2h/day. In turn, boys reported playing games on a computer, games console (like PlayStation, Xbox, GameCube), tablet, smartphone, or other electronic device (not including moving or fitness games) on average 2.9 ± 1.8h/day, while girls, 2.1 ± 1.4h/day on average. While boys reported using electronic devices such computers, tablets, or smartphones for other purposes, for example, homework, emailing, Facebook, Instagram, chatting, surfing the Internet for 2.1 ± 1.6h/day on average, girls reported 1.8 ± 1.0h/day on average. The only statistically significant difference between the compared groups in terms of specific screen time was found for playing games (*p* = 0.008).

A total of 63% of children met age-appropriate sleep guidelines. The mean hours of sleep per night on a weekday was 9.0 ± 0.9h/day and did not differ statistically between girls and boys.

The mean average of selected school skills can be found in Table 1. In general, in all tests where the pace mattered, boys obtained better results, completing the tasks faster than girls (see: visual-spatial functions and visual-auditory integration). Whereas, girls performed better in tests where accuracy was important rather than speed (visual-spatial functions, perceptual-motor integration, auditor and linguistic functions). There were significant differences between girls and boys in the perceptual-motor integration(girls 7.4 ± 7.6 vs boys 5.3 ± 2.0, *p* = 0.013) and the auditory and linguistic functions (7.2 ± 2.5 vs. boys 6.3 ± 2.3, *p* = 0.034).

The analysis conducted for 8–9-years-old children revealed that several factors influenced the three selected academic skills: visual-spatial functions, visual-auditory integration, and perceptual-motor integration. These factors included MVPA, sex, not meeting the screen time guidelines, and not sufficient sleep time, as shown in Table 2. Boys were characterised by a significantly better time of the visual-spatial functions compared to girls (B = -0.20, *p* = . 049).

**Table 2:** Multivariable nonlinear regression for selected academic skills – visual-spatial functions, visual-auditory integration, perceptual-motor integration, auditory and linguistic functions in relation to sex, MVPA screen time and sleep time factors duration in children 8/9-year-old (adjusted)

	Visual-spatial functions(sec)			Visual-auditory integration(sec)			Perceptual-motor integration(pts)		
	B (SE)	95% CI	*p*	B (SE)	95% CI	*p*	B (SE)	95% CI	*p*
MVPA (days/week)	-0.09 (0.06)	(-0.20, 0.03)	.140	**-0.07 (0.03)**	**(-0.13, -0.03)**	**.040**	-0.003 (0.02)	(-0.04, 0.04)	.901
**Sex**									
Female	ref			ref			ref		
Male	**-0.20 (0.10)**	**(-0.40, -0.0006)**	**.049**	-0.09 (0.05)	(-0.20, 0.01)	.093	**-0.14 (0.03)**	**(-0.21, -0.07)**	**.000**
**Screen time categories**									
≤ 2h/day	ref			ref			ref		
> 2h/day	0.03 (0.12)	(-0.22, -0,27)	.826	-0.07 (0.07)	(-0.20, 0.07)	.329	**-0.09 (0.04)**	**(-0.18, -0.005)**	**.040**
**Sleep time categories**									
< 9h/day	-0.10 (0.10)	(-0.31, 0.10)	.334	**0.12 (0.06)**	**(0.004, 0.23)**	**.042**	-0.0003 (0.04)	(-0.07, 0.07)	.993
≥ 9h/day	ref			ref			ref		

B – parameter, *SE* standard error, *CI* confidence interval. Bold indicates statistically significant results

A significant association was observed between MVPA and the visual-auditory integration. Higher levels of MVPA were associated with better scores in visual-auditory integration (B = -0.07, *p* = 0.040). Additionally, boys who slept less than 9 h per day had significantly lower scores in visual-auditory integration compared to the children slept 9h or more per day (B = 0.12, *p* = 0.042).

The most important predictors for the perceptual-motor integration were found to be sex and screen time categories. Compared to girls, boys had a significantly better perceptual-motor integration scores (B = -0.14, *p* = 0.000). Furthermore, students who spent more than 2h per day watching TV scored worse (pts) in perceptual-motor integration (B = -0.09, *p* = 0.040) compared to those spending less than 2h per day.

## Discussion

The results of previous and converging studies indicate that knowledge about the role of PA in the context of maintaining and improving health is still very poorly disseminated, and the resulting applications are not sufficiently implemented in everyday practice [9]. This inadequacy translates into low levels of PA in school-aged children and adolescents (Global Matrix, GM 2.0; GM 3.0). Furthermore, for young people, the indicated state is not improved by the progressive access to electronic devices, which may be conducive to a sedentary lifestyle and, consequently, negatively affect their cognitive development and lower school achievement, as indicated in studies conducted by other researchers [22]. For this reason, given the current state of knowledge, our study aimed to investigate how PA, screen time, and sleep duration influence the level of school competence among young individuals of both sexes. With these goals in mind, we established specific assumptions to guide our research. The results and conclusions obtained are presented in the following paragraphs.

Our research revealed that a significant majority of children in the early school age group, specifically 92% overall and 94% of boys, participate in PA for less than the recommended 60min per day. These findings indicate that the level of PA engagement among the surveyed students falls below the standards set by the WHO [12]. This outcome aligns with previous Polish analyses conducted in 2021 [40]. Additionally, the same study found that a substantial 78% of children exceed the recommended amount of time limit of up to 2h a day [13]. These findings are consistent with the results of other studies conducted on Polish children [40, 41].

Interestingly, electronic screen devices are not solely used for entertainment purposes by students [42] but have also been extensively integrated into education practices [41, 43]. At the same time, a large percentage of children (37%) were found to have insufficient sleep time, with the reference time range suggesting 9 to 11–12h of sleep per day [13, 36]. These results are concerning, as regular, age- and health-adapted physical activity plays a crucial role in the multidirectional development of students, encompassing physical, mental, and social aspects in the context of a dynamically developing digital landscape [4, 44]. Similarly, modifiable lifestyle behaviours such as PA, screen time, and sleep time have been identified as important factors influencing emotional health and academic success [45, 46]. Conversely, the combination of inadequate PA, insufficient sleep, and excessive screen time can lead to obesity and reduced cardiorespiratory fitness [47]. Convergent analyses also indicate that excessive screen time among school children can lead to concentration disorders, eye damage [48], negatively affect the development of fine motor skills [49], and if prolonged before bedtime, negatively impact sleep and consequent body regeneration [50]. These conclusions are particularly disturbing when considering data showing that 25% of parents allow their children to use smartphones or tablets while eating meals, and 18% provide their children with mobile devices to aid in falling asleep [51].

Based on the analysis of the collected data on the independent variables (level of PA, screen time, sleep time) and dependent variables (results in tests assessing for the psychological and pedagogical diagnosis of specific reading and writing difficulties) [37], it was observed that reduced sleep duration (which affects the accuracy of decision-making and memory consolidation) and lower levels of PA have a negative impact on the performance of rapid naming tests. These tests assess the speed at which familiar visual stimuli are verbally labeled, and a deficit in this ability is considered as an important indicator of precise information coordination, particularly in the diagnosis of developmental dyslexia [37]. Furthermore, Wolf, Bowers, and Biddle [52] suggest that the experience of a particular ability imitates the process of reading, making these rapid naming tasks useful for predicting or identifying children at risk for reading difficulties. Moreover, the specific research findings regarding the negative relationship between sleep duration, PA, and the coordination of information is consistent with analyses conducted in the neurocognitive field [53, 54] and studies on decision-making skills [55]. These findings further support the assumption regarding compliance with the recommendations regarding the appropriate amount of sleep [13], as it contributes to the acceleration of post-exercise regeneration, including that related to the child’s participation in PA.

Additional findings from the study revealed a negative correlation between screen time and performance in the visual-auditory integration test, which assesses the ability to integrate and transfer information across different sensory modalities. Moreover, the results indicated that girls outperformed boys in tests related to phonological memory (the ability to remember and reproduce the order of sounds in words) and visual-auditory integration. The result can be identified – also demonstrated in our research – by a significantly greater range of time spent by schoolboys on activities related to playing games on computers, consoles, tablets, or smartphones compared to schoolgirls. The results are consistent with previous research on screen time and cognitive difficulties, exacerbated by reduced PA [20], as well as lifestyle behaviours with student performance in education [24].

Researchers emphasise that for proper development, children need to engage their senses and experience the world in a multisensory manner. Limiting exposure to diverse stimuli, as observed with excessive screen time, can negatively affect the development of neural structures of the brain [56]. This, in turn, may lead to difficulties in mastering reading and writing skills, establishing social connections, and in extreme situations, contributes to addiction [57]. This issue is significant as PA, a fundamental activity for promoting health, is increasingly being replaced by mass media usage as technology advances and children’s media consumption patterns change over time [58].

Notably, a British study *Children and Parents: Media Use and Attitudes* [59] found that 35% of children aged around 7 owned smartphones, and 66% 6-year-olds regularly played games for a minimum of 7.5h a week. A similar project, Zero to Eight research series conducted by the American organisation Common Sense, showed that 98% of children under the age of 8 have access to a mobile device at home, and 42% own such a device [58].

In response to the growing issues regarding the safety and well-being of children amidst widespread use of screen devices in life and education [43], various organisations and authorities have introduced recommendations and initiatives. In 2019, the WHO emphasised the importance the regular need for PA and adequately long and comfortable sleep for children [60]. Similarly, in the United States, recommendations have been made to allocate designated time for meals and transportation without media use, as well as media-free areas within the home [61].

The concept of “digital hygiene” has also emerged, focusing on mitigating excessive screen time and promoting responsible digital device use. The concept applies not only to children but also to parents, who are encouraged to educate themselves and provide assistance in complying with clear rules for using digital devices [43]. At the same time, in certain countries, measures have been taken out of concern for children’s mental health, such as implementing bans on the use of mobile phones in schools. Recent reports from schools in southern Australia highlight the introduction of a mobile phone ban in schools to reduce online bullying and the spread of anti-social behaviour [source: https://www.mamamia.com.au/mobile-phone-ban-australia/].

Based on the findings of our research, several key points should be emphasised. First, there is a clear insufficiency in PA levels and sleep among the surveyed children, coupled with excessive screen time. It is important to note that this term “sitting in front of screens” seems to be too general, because many children spend their time using more than one source of blue light, such as watching TV while simultaneously being active on social media [11]. Second, the sedentary lifestyle, which has been recognised as early as 2010 as the fourth largest risk factor for mortality in the world [10], has a detrimental impact on human cognitive functioning, ultimately affecting the quality of children’s academic achievements. Therefore, we would like to draw attention to the legitimacy of promoting these activities that affect the multidirectional development of children, undertaken not only by education managers or physical education teachers themselves who can encourage students to act and compete in sports, but also by active families – the closest environment for a child’s development. Families play a vital role in establishing healthy habits, promoting pro-health behaviours, and providing opportunities for active leisure time. Additionally, it is essential to create environments that support and facilitate active forms of engagement before, during (e.g., school breaks), and after students’ school activities. Promoting and prioritising these activities can contribute to the overall well-being and development of children.

The present study acknowledges several limitations that should be considered. These limitations of the study include: 1) its cross-sectional design, which limits drawing and conclusions regarding causality, 2) the small sample size and the non-probabilistic (non-random) sampling, which does not allow us to make inferences for all children, 3) the self-reported questionnaires for PA, screen time, and sleep time.

## Conclusions

This study adds to the growing body of evidence linking PA, reducing screen time and sufficient sleep time to obtain better academic skills in children. Majority of the examined children failed to meet MVPA and screen time recommendations, while over half of them met age-appriopriate sleep guidelines. School and the family have to focus not only on children’s academic achievements, but also on their wellness and health. Future research efforts should be focused on creating and implementing integrated wellbeing interventions based on the benefits of being active, less sedentary, and getting enough sleep.

## Data Availability

The datasets used and/or analysed during the current study are available from the corresponding author on reasonable request.

## Abbreviations

PA: Physical activity
MVPA: Moderate to vigorous intensity physical activity
WHO: World Health Organization
OPA: Overall physical activity
SB: Sedentary behaviours
BMI: Body mass index
HBSC: Health Behaviour in School-aged Children
M: Mean
SD: Standard deviation
GM: Global Matrix
